# Microutrophin expression in dystrophic mice displays myofiber type differences in therapeutic effects

**DOI:** 10.1371/journal.pgen.1009179

**Published:** 2020-11-11

**Authors:** Glen B. Banks, Jeffrey S. Chamberlain, Guy L. Odom

**Affiliations:** 1 Department of Neurology, University of Washington, Seattle, Washington, United States of America; 2 Department of Medicine, University of Washington, Seattle, Washington, United States of America; 3 Wellstone Muscular Dystrophy Specialized Research Center, University of Washington, Seattle, Washington, United States of America; 4 Department of BioChemistry, University of Washington, Seattle, Washington, United States of America; The Jackson Laboratory, UNITED STATES

## Abstract

Gene therapy approaches for DMD using recombinant adeno-associated viral (rAAV) vectors to deliver miniaturized (or micro) dystrophin genes to striated muscles have shown significant progress. However, concerns remain about the potential for immune responses against dystrophin in some patients. Utrophin, a developmental paralogue of dystrophin, may provide a viable treatment option. Here we examine the functional capacity of an rAAV-mediated microutrophin (μUtrn) therapy in the *mdx*^*4cv*^ mouse model of DMD. We found that rAAV-μUtrn led to improvement in dystrophic histopathology & mostly restored the architecture of the neuromuscular and myotendinous junctions. Physiological studies of tibialis anterior muscles indicated peak force maintenance, with partial improvement of specific force. A fundamental question for μUtrn therapeutics is not only can it replace critical functions of dystrophin, but whether full-length utrophin impacts the therapeutic efficacy of the smaller, highly expressed μUtrn. As such, we found that μUtrn significantly reduced the spacing of the costameric lattice relative to full-length utrophin. Further, immunostaining suggested the improvement in dystrophic pathophysiology was largely influenced by favored correction of fast 2b fibers. However, unlike μUtrn, μdystrophin (μDys) expression did not show this fiber type preference. Interestingly, μUtrn was better able to protect 2a and 2d fibers in *mdx*:utrn^-/-^ mice than in *mdx*^*4cv*^ mice where the endogenous full-length utrophin was most prevalent. Altogether, these data are consistent with the role of steric hindrance between full-length utrophin & μUtrn within the sarcolemma. Understanding the stoichiometry of this effect may be important for predicting clinical efficacy.

## Author summary

Duchenne muscular dystrophy (DMD) is a severe muscle wasting disorder caused by mutations in the dystrophin gene. Utrophin is structurally similar to dystrophin and can potentially be utilized to prevent muscle necrosis in preclinical models of DMD. Consequently, utrophin-mediated therapies are a primary target for treating DMD, particularly as it may circumvent immune responses to dystrophin-mediated therapies. One promising therapeutic option is to utilize recombinant adeno-associated viral vectors (rAAV) to deliver a rationally designed miniaturized utrophin (μUtrn) to striated muscles to prevent necrosis. Here, we found that rAAV-μUtrn can profoundly prevent skeletal muscle necrosis in the *mdx*^*4cv*^ mouse model of DMD. μUtrn was also able to replace many functions of dystrophin at the neuromuscular synapse and myotendinous junctions. However, we provide several lines of *in vivo* evidence that steric hindrance between μUtrn and the endogenous full-length utrophin on the sarcolemma impacts the longevity of the therapy in a muscle fiber-type selective manner. Understanding the stoichiometry of this effect may be important for predicting clinical efficacy.

## Introduction

**Fig 1:**
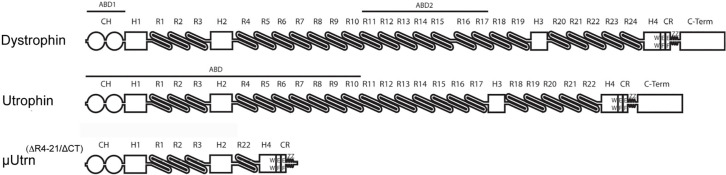
Domain structure of dystrophin, utrophin and μUtrn. Shown is an illustration depicting the major functional domains of full-length, (427 kDa) dystrophin, (400 kDa) utrophin, and (130 kDa) microutrophin (μUtrn). Dystrophin contains two actin-binding domains [ABD1, which has 2 calponin-homology domains (CH), and ABD2), while the single ABD of utrophin is continuous through spectrin-like repeat 10. The central rod domain of dystrophin is composed of 24 spectrin-like triple-helical elements or repeats (R) while utrophin contains 22 repeats, both proteins carry 4 hinge (H) domains. Toward the carboxy-terminus a WW domain within hinge 4 together with a cysteine rich (CR) domain composed of 2 EF-hands, and a Zinc finger domain (ZZ), form a binding domain for beta-dystroglycan. The Carboxyl-terminal domain (C-term) provides binding sites for the syntrophins and dystrobrevins. Micro-utrophin lacks repeats 4–22 as well as the CT domain (Odom, 2010).

Duchenne muscular dystrophy (DMD) is a severe muscle wasting disorder caused by mutations in the dystrophin gene [1, 2]. Mechanically, dystrophin functions in muscle akin to a large molecular spring that connects the cytoskeleton via actin to the dystrophin-glycoprotein complex (DGC) within the sarcolemma [3–8]. As such, muscle membranes in DMD are highly susceptible to contraction-induced injury and hypoxic stress after mild exercise [9–17]. Furthermore, the neuromuscular junctions in the *mdx* mouse model of DMD fragment upon skeletal muscle necrosis, and have fewer and shallower postsynaptic folds than wild-type muscles [18–20]. The folding is also reduced at the myotendinous junctions, which is a prominent site for force transfer and injury in some DMD patients [21–26]. The dystrophin protein contains an N-terminal actin binding domain, a large central rod domain, a cysteine rich region critical for the association with β-dystroglycan[27], and a C-terminal domain [28, 29] (**Fig 1A**). The large central rod domain includes 24 spectrin-like repeats that interact with the membrane [30], F-actin [31], localize nNOS to the sarcolemma [32, 33], and guide peripheral microtubules [34]. The central rod domain also includes 4 hinge regions that contain high concentrations of proline residues [8]. As an X-linked disorder, DMD is potentially amenable to dystrophin replacement gene therapies [35, 36]. In fact, rational design of miniaturized dystrophins for gene therapy using rAAV has proven affective at mitigating the dystrophic pathophysiology in various mammalian models of DMD [29, 32, 37–41]. Clinical trials that utilize different forms of μDys have reported varied success at an early stage of treatment, but none are anticipated to fully restore normal muscle function[42]. One concern regarding DMD clinical therapeutic development is the possibility that dystrophin may be recognized by the immune system as a neo-antigen in some DMD patients [43–46]. As such, we are also interested in the therapeutic capacity of the dystrophin paralogue, utrophin [44, 47].

Utrophin is structurally similar to dystrophin in that it contains an N-terminal actin-binding domain, a large central rod domain containing 22 spectrin-like repeats with 4 hinges, a cysteine rich region and a C-terminal domain [28] (Fig 1). Despite the structural similarities, utrophin differs from dystrophin in a variety ways, such as having a single actin-binding domain that extends from the N-terminus through to spectrin-like repeat 10; in contrast dystrophin has a secondary actin binding domain (ABD2, Fig 1) [48]. Interestingly, utrophin also displays differing molecular contact responses to actin relative to dystrophin, affecting rotational dynamics, resulting in increased actin resilience [48, 49]. Indeed, it has been hypothesized that one continuous actin binding site may display less elasticity toward contractile responses via the actin-utrophin-sarcolemma linkage to muscle stretches relative to dystrophin[50]. Whether this could be exacerbated *in vivo* considering the structure of micro-utrophin^(ΔR4-R21/ΔCT)^ (μUtrn), having a continuous ABD that constitutes for ~65% of the entire micro-protein, is not known. To this end, it has also been found that μUtrn is as effective *in vitro* at regulating actin dynamics as full-length dystrophin as determined by time-resolved phosphorescence anisotropy[51]. A further contrast from dystrophin in the central rod domain has been demonstrated downstream of the dystrophin ABD2, where utrophin lacks the binding domains associated with nNOS restoration to the sarcolemma or the ability to guide microtubules[34, 52]. Despite these differences, transgenic expression of full-length utrophin can prevent muscle necrosis in sedentary *mdx* mice, thus making it a promising candidate for treating DMD patients, independent of the type or placement of the dystrophin mutation [44, 53]. The μUtrn construct contains essential functional elements of the native utrophin, namely N-terminal actin-binding domain, four spectrin-like triple helices of the central rod domain (R1-R3 + R22), two hinges (H2 & H4), and the CR domain enabling binding to beta-dystroglycan[27](i.e. ΔR4-R21/ΔCT), enabling DGC assembly for localization at the sarcolemma [7, 9, 54–56]. Indeed, repeat administration of TAT-μUtrn has been shown to mitigate the pathophysiology of *mdx* and *mdx*:*utrn*^-/-^ double knockout (*dko*) mice [57, 58]. Similarly, rAAV-mediated delivery of uUtrn prolongs the lifespan and mitigates the skeletal muscle dystrophic pathophysiology of *dko* mice [47] and in the canine model of DMD[46]. Although μUtrn therapy for *dko* mice clearly provided a benefit with improvement and stabilization of the histopathology with improved functional capacity, and lifespan extension; animals were not “cured” per se as a result of the treatment. Importantly, in a recent study utilizing rAAV9-mediated μUtrn delivery to severely affected *D2/mdx* mice, a demonstrated benefit of the cardiac phenotype was reported using cine-MRI for indices such as stroke volume and ejection fraction, providing the first evidence of a functional cardiac benefit with μUtrn upregulation [59].

A potential compounding issue with a μUtrn therapeutic for DMD is whether endogenous utrophin could influence the expression, localization, and inherent functional capacity of μUtrn, with utrophin being generally present at low levels on the sarcolemma and also highly concentrated within the neuromuscular and myotendinous junctions [60–62]. The design of μUtrn was based on the first generation μDys^(ΔR4-R23/ΔCT)^ capable of being packaged within AAV, where the μDys cDNA contained the N-terminal actin binding domain, a small central rod domain containing hinges 1, 2, and 4, with four spectrin-like repeats, and the cysteine rich region [47, 63] (Fig 1A). Of note, μUtrn also includes a conserved polyproline site within hinge 2, that when present in μDys^(ΔR4-R23/ΔCT)^, led to myotendinous strain injury, ringed myofiber formation, fragmentation of the neuromuscular synapses, and elongation of the synaptic folds in a subset of muscles in *mdx* mice [19, 26, 39]. Similar deleterious effects might be caused by the inclusion of hinge 2 in μUtrn. Therefore, in addition to histopathological and physiological assessment, this study was further aimed at evaluating neuromuscular and myotendinous junctions in *mdx*^*4cv*^ mice treated with AAV-mediated delivery of μUtrn.

## Results

Fig 1 shows the domain structure of the μUtrn^(ΔR4-R21/ΔCT)^ protein used in this study as well as the structures of full-length dystrophin and utrophin [63]. The 1,160 residue, 130 kDa μUtrn has an analogous structure to a highly functional first-generation μDys^(ΔR4-R23/ΔCT)^ we described previously [47, 63], currently being tested in phase 1/2 clinical trials (NCT03375164). Both micro-proteins carry 4 spectrin-like repeats in the central rod domain, but contain the entire N-terminal actin-binding and the cysteine-rich (CR) dystroglycan-binding domains, enabling essential binding to F-actin, beta-dystroglycan, and assembly of the dystrophin-glycoprotein complex (DGC) at the sarcolemma (except for nNOS) [7, 9, 52, 54–56, 64]. The μUtrn protein also lacks the C-terminal (CT) domain, which can be deleted from utrophin or dystrophin with minimal impact on function [47, 63, 65]. The absence of the CT domain also enables endogenous Utrn to be distinguished from μUtrn using antibodies that recognize the CT domain. Both proteins are detected with antibodies against the N-terminal domain of Utrophin A. In some studies we added a N-terminal flag epitope for exclusive detection of μUtrn. Expression vectors for μUtrn were prepared in rAAV6 as previously described [37, 47, 66].

### Histopathlogy of mdx^4cv^ muscles treated with μUtrn

**Fig 2:**
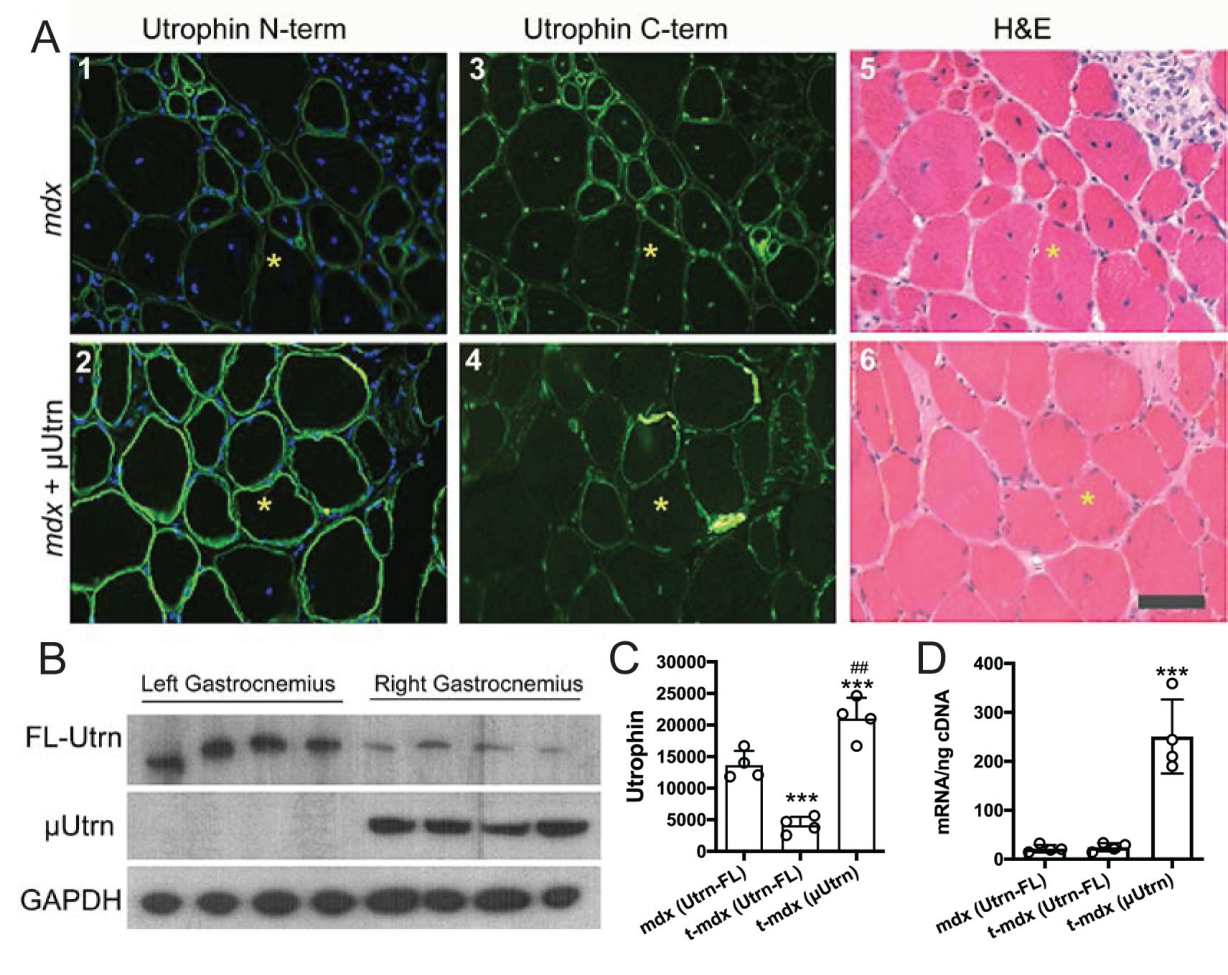
μUtrn expression mitigates dystrophic pathology. (A.) Adjacent sections of *mdx*^*4cv*^ muscles and *mdx*^*4cv*^ muscles directly injected with rAAV6-CK8-μUtrn. Scale bar = 50 μm. (B.) Western blot showing full length utrophin and μUtrn in untreated (left) and treated (right) gastrocnemius muscles (n = 4). GAPDH is the loading control. (C.) Quantitation of utrophin signal intensity from the Western blots. ***P < 0.001 compared to utrophin full-length in the treated muscles. (D.) Quantitation of full length utrophin and micro-utrophin mRNA by qRT-PCR. ***P < 0.001 compared to utrophin full-length in treated and untreated muscles.

Utrophin is expressed in new and maturing skeletal muscles during embryonic development in humans, and in mice this extends to ~2-weeks postnatal. As the muscle matures, utrophin expression is reduced and is ultimately replaced by dystrophin on the sarcolemma of normal muscles, but remains concentrated at the neuromuscular and myotendinous junctions. In the absence of dystrophin in DMD, utrophin appears to maintain this cadence where the expression is reduced as the muscle matures. However, without dystrophin expression to replace utrophin, the muscles become highly susceptible to contraction-induced injury. Muscle necrosis and the resulting inflammatory response activates resident satellite cells to regenerate myofibers leading to the re-establishment of utrophin at the sarcolemma of the maturing fibers. This cycle of utrophin expression continues for as long as the satellite cells are capable of replacing the lost muscle and leads to a patchwork of utrophin expression in the *mdx* muscles that are reflective of the stage of regeneration and maturation (Fig 2A). It is well known that slower muscle fiber types have higher levels of residual utrophin on the sarcolemma in DMD and are more resistant to the dystrophic pathophysiology[67–69]. To examine the effects of expressing human μUtrn in dystrophic mice, rAAV6-CMV-μUtrn (2x10^10^ vector genomes (vg) was injected into the right gastrocnemius and tibialis anterior (TA) muscles of 1 week old *mdx*^*4cv*^ mice, with the sham-injected contralateral muscles as controls. At 3 months post-injection the tissues were harvested, and adjacent cryosections were immunostained with N-terminal utrophin antibodies to detect both μUtrn and endogenous full-length utrophin (FL-Utrn), and with C-terminal utrophin antibodies to exclusively detect FL-Utrn. Sections were also stained with hematoxylin and eosin to detect inflammatory pathology and centrally-nucleated myofibers, a hallmark of muscle regeneration (Fig 2A, panels 5, 6; S1 Fig).

Consistent with previous studies [70], upregulated FL-Utrn was detectable in most myofibers within the dystrophic environment of untreated *mdx*^*4cv*^ mouse gastrocnemius muscles (Fig 2A, panels 1, 3). Approximately 76% of the myofibers in untreated *mdx*^*4cv*^ gastrocnemius muscles exhibited central nuclei (a marker of muscle regeneration, S1A Fig) and extensive areas of mononuclear cell infiltrates (Fig 2A, panel 5). Intense N-terminal immunostaining of Utrn was observed in the rAAV6-CMV-μUtrn-treated gastrocnemius muscles. Only ~3% of the Utrn-positive myofibers in treated muscles displayed centrally-located nuclei (S1 Fig). Importantly, the muscle fibers expressing uUtrn also contained low levels of FL-Utrn as demonstrated by the antibody that recognizes the C-terminus of FL-Utrn and is absent in the uUtrn.

μUtrn-treated gastrocnemius muscles also displayed an improved morphological appearance and significantly fewer regenerating myofibers, as assessed by immunostaining for “developmental” (embryonic or neonatal) myosin heavy chain (18% positive myofibers compared with 67% for controls; (Fig 2A, panels 5 and 6; S1 Fig). This reduction in myofiber regeneration presumably accounts for the reduced full-length (FL) utrophin expression observed by both western analysis and immunostaining (Fig 2B and 2C), as regenerating myofibers have been shown to express elevated utrophin levels [71]. In contrast to the reduction in protein levels in treated mice, we found no difference in FL-Utrn mRNA levels between treated and untreated *mdx*^*4cv*^ gastrocnemius muscles (Fig 2D). As anticipated, the mRNA levels of CMV-driven μUtrn were found to be much higher than the full-length transcripts in treated muscles (*P* < 0.001; Fig 2D). Expression of μUtrn in *mdx*^*4cv*^ gastrocnemius muscles is thus sufficient to substantially ameliorate the dystrophic pathology.

### Physiological performance of mdx^4cv^ muscles treated with μUtrn

To assess the functional capacity of μUtrn to ameliorate physiological deficits in *mdx*^*4cv*^ muscles, we analyzed contractile properties of the treated TA muscles *in situ*, followed by assessment of morphology and utrophin expression.

**Fig 3:**
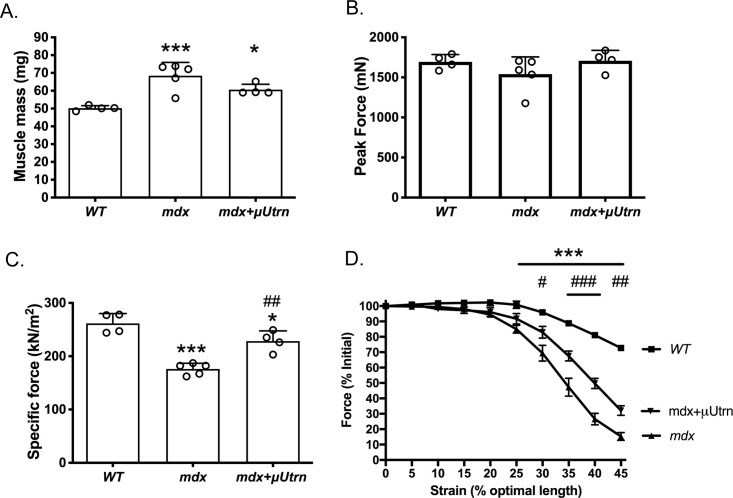
*In situ* physiological assessment of tibialis anterior muscles following vector treatment. (A.) Mean +/- S.D. tibialis anterior muscle mass. (B.) Mean +/- S.D. peak tetanic force production. (C.) Mean +/- S.D. specific force production. (D.) Peak tetanic force production after increasing strain. *P < 0.05 and ***P < 0.001 compared to wild-type. ^#^*P* < 0.05, ^##^*P* < 0.01 and ^###^*P* < 0.001 compared to *mdx*^*4cv*^.

We examined several physiological parameters known to be altered in *mdx*^4cv^ TA muscles: *(1) Muscle Mass*: As previously reported muscle mass increases significantly in *mdx* mice and this is thought to be partially responsible for maintaining peak force production of *mdx* muscles [72, 73]. We confirmed the mass increase of *mdx*^*4cv*^ TA muscles, and found that μUtrn expression partially reduced this increase (Fig 3A). *(2) Peak Force Production*: The increased muscle mass maintains peak force production in *mdx*^*4cv*^ mice at this age, and was also unchanged in limbs treated with uUtrn. (Fig 3B). *(3) Specific Force Production*: This parameter is typically reduced in *mdx* muscles [37, 73], and we found it to be reduced by about 35% in *mdx*^*4cv*^ TA muscles; μUtrn-treatment restored about 60% of this deficit (P<0.05; Fig 3C). *(4) Susceptibility to Eccentric Contraction-Induced Injury*: Since *mdx*^*4cv*^ muscles are known to be susceptible to contraction-induced injury [37, 73], we used an *in situ* strain protocol to determine whether μUtrn treatment would ameliorate this damage. When subjected to increased strain, peak tetanic force production of *mdx*^*4cv*^ TA muscles decreased significantly compared to the smaller decrease in wild type TAs. In contrast, μUtrn-treated dystrophic muscles exhibited significant protection against this perturbation (Fig 3D), albeit to a degree notably less than in wild type muscles or in previous studies with micro-dystrophin ([26], & S3 Fig).

Immunostained cryosections of TA muscles at 3 months post-injection revealed that ~60% of the total myofibers were μUtrn-positive, based on their much more intense N-terminal Utrn immunostaining (see Fig 2A, panel #2 vs. #1). The physiological data we measured thus represents the combined attributes of about 40% of the total fibers that contain little to no μUtrn (but variable levels of endogenous utrophin), and about 60% of the total fibers that contain variable μUtrn levels due to differences in fiber-to-fiber transduction.

### Myofiber type differences in μUtrn expression

**Fig 4:**
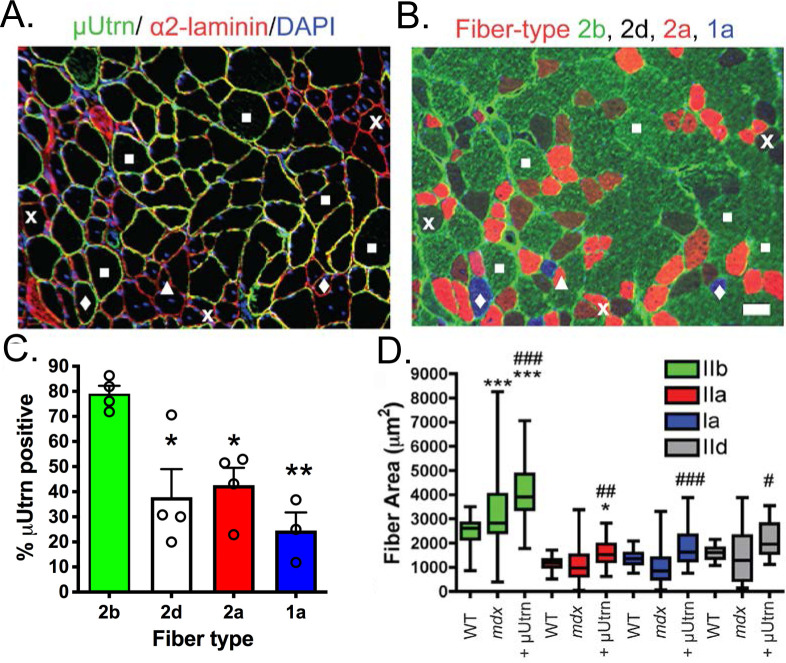
μUtrn expression mitigates dystrophic pathology and demonstrates fiber-type preferences in gastrocnemius muscles. (A) Immunostaining for utrophin A indicating high level expression of (green), laminin A (red), and nuclei (blue). (B) Adjacent sections of μUtrn treated *mdx*^*4cv*^ muscles with corresponding fiber typing. Symbols provide examples of 1a, 2a and 2d/x fibers expressing high levels of μUtrn. The white squares, “x’s”, triangles, and diamonds indicate type 2b, 2d/x, 2a, 1a fibers respectively. Scale bar = 50 μm. (C.) Quantification of the proportion of fiber types expressing μUtrn (Mean +/- S.D.). (D.) Myofiber area as indicated by fiber type. Box and whiskers plots displaying median +/- 75% fiber area in wild-type, *mdx*^*4cv*^ and *mdx*^*4cv*^ treated skeletal muscles. μUtrn expression leads to myofiber hypertrophy in all muscle fiber types. Bars show the mean +/- S.D., (N = 4). *P < 0.05 and **P < 0.01 relative to wt ^#^*P* < 0.05, ^##^*P* < 0.01, ^###^*P* < 0.001 relative to untreated *mdx*^*4cv*^.

A fundamental question of this study is whether expression of full-length utrophin functions synergistically with the highly truncated uUtrn or whether there is a potential for steric hindrance. Considering full-length utrophin is expressed at higher levels in type 1a, 2a and 2d fibers when compared to type 2b fiber types we examined whether there is a fiber-type expression pattern of μUtrn in treated muscles. μUtrn was more favorably expressed in the fast 2b fiber types (79% μUtrn-positive) when compared with Ia (25% positive), 2a (38% positive) and 2d (43% positive) fibers at 3 months post-injection (Fig 4A–4C). Interestingly, μUtrn-positive myofibers displayed a further increase in cross-sectional area beyond that observed in *mdx*^*4cv*^ TA muscle, regardless of fiber type (Fig 4D).

To explore whether the μUtrn fiber-type expression differences may have resulted from properties of the CMV promoter/enhancer elements, we tested a vector carrying the muscle-specific regulatory cassette CK8e (kindly provided by Dr. Stephen D. Hauschka). Initially, 2 week-old *mdx*^*4cv*^ mice were intravenously (IV) infused with 2x10^14^ vg/kg of rAAV6-CK8e-Flag-μUtrn and muscles were examined 2 weeks post-injection (at 1-month of age). This experimental design focused on early stages of transgene expression, and the onset of *mdx*^*4cv*^ mouse muscle pathology [74]. As revealed by immunostaining against the N-terminal FLAG epitope, μUtrn was found on the sarcolemma of approximately 88% of all muscle fiber types (S2A–S2C Fig; gastrocnemius muscle shown). This finding suggested that rAAV6-CK8e driven expression of μUtrn has a similar tropism for all four myofiber types. A second cohort of mice intravenously infused with rAAV6-CK8e-Flag-μUtrn was examined at a later time point (3 months of age). If uUtrn is not protecting the skeletal muscle, the muscle fibers deteriorate and regenerate leading to loss of the therapeutic cDNA and newly formed uUtrn negative myofibers. Similar to the earlier study with IM injection of rAAV6-CMV-μUtrn, we found that with IV delivery, μUtrn was expressed within a significantly greater number of type 2b myofibers compared with type 1, 2a and 2d fibers (P < 0.001; S2D–S2F Fig). Thus, the preferential expression of μUtrn in the fast 2b fibers at later time points was similar whether the CK8e (S2D–S2F Fig) or the CMV (S2G–S2I Fig) regulatory cassettes were used. Importantly, the number of myofibers expressing μUtrn at 3 months of age was significantly reduced from the earlier time points demonstrating the uUtrn was less able to protect type 1a, 2a and 2d fibers from dystrophy when compared to the type 2b fibers (S2D–S2F Fig).

As a further control, *mdx*^*4cv*^ mice were intravenously injected with rAAV6-CK8e-μDys and analyzed 8 months later. In contrast with the μUtrn studies, expression of μDys was maintained in the vast majority of myofiber types even at 8 months, leading to a significant functional improvement of the muscles (S3 Fig). We also note that our original hinge2-μDys[75, 76] displayed stable expression for up to one year in DMD patients[77], and that a different μDys vector, rAAV6-CK8e-μDys5, showed stable expression in all striated muscles for more than 27 months in *mdx*^4cv^ mice [78].

**Fig 5:**
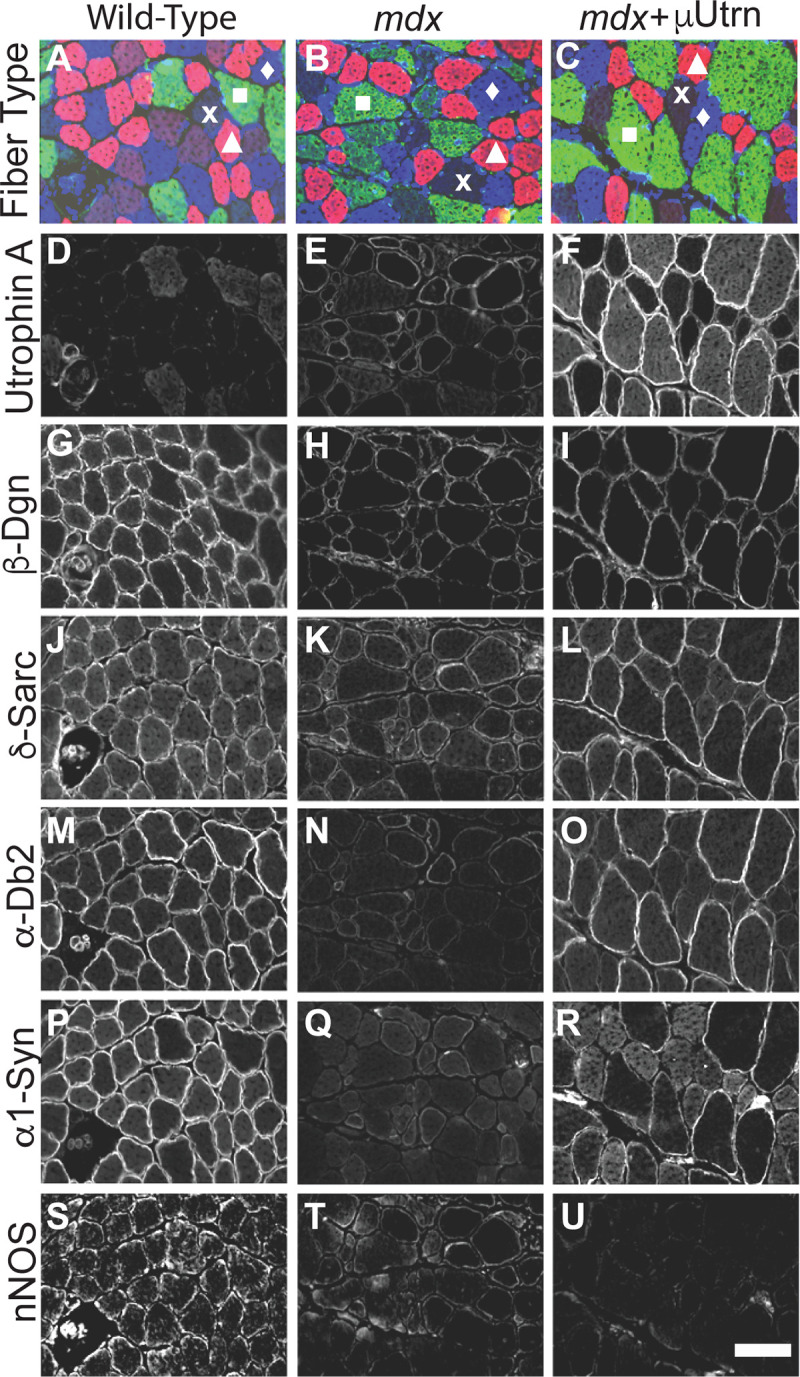
Restoration of DGC components α-dystrobrevin-2 and α_1_-syntrophin localization to the dystrophin-glycoprotein complex by μUtrn expression is fiber type selective. Scale bar = 100 μm.

Considering uUtrn co-exists on the sarcolemma with the full-length utrophin and that full-length utrophin is found at higher levels in type Ia, 2a, and 2d fibers (Fig 5 for example, and [71]) that are less protected by uUtrn, these results raised the possibility that endogenous utrophin expression could adversely impact the therapeutic potential of μUtrn. To evaluate μUtrn expression in the absence of endogenous utrophin we examined the gastrocnemius muscles of *mdx*:*utrn*^*-/-*^ (*dko*) mice from a previous study [47]. These mice had received an intravenous injection of 2.5x10^14^ vg/kg of rAAV6-CMV-μUtrn, at 3 weeks of age and were analyzed at 4 months of age. We found no preference toward μUtrn expression in the fast 2b fibers when compared with the 2a or 2d fiber types (S2J–S2L Fig). The status of type 1a fibers in this study is uncertain because there were very few type 1a myofibers in the *dko* gastrocnemius and only ~6% of these had detectable μUtrn (S2J–S2L Fig).

### μUtrn expression in mdx^4cv^ muscle fibers affects costamere structure

Costameres are rib-like sarcolemmal structures juxtaposed over the Z and M lines of myofibers, and are the primary sites where cytoskeletal proteins link elements of the contractile apparatus to integral and peripheral membrane protein complexes [79, 80]. Costameres can also be oriented parallel to the myofiber longitudinal axis, resulting in a rectilinear structural lattice. Historically, dystrophin represents the first costameric protein found to be associated with muscular dystrophy, displaying periodic association with costameres running transversal to the long axis of the myofiber [79, 81]. Interestingly, in *mdx* myofibers, the up-regulated FL-Utrn only partially compensates for the absence of dystrophin, leading to a weakened linkage between the contractile apparatus and the sarcolemma [79, 82–84]. To examine the effects of μUtrn on *mdx*^*4cv*^ costameres, gastrocnemius muscles were removed from 3-month old FLAG-μUtrn-treated and control *mdx*^4cv^ mice and cryosections were immunostained for endogenous utrophin A (using a utrophin C-terminal antibody), or for μUtrn (using a FLAG antibody). In longitudinal cryosections endogenous utrophin A was found within a standard ~2.2 μm costameric lattice, as well as between the costameric striations (S4A Fig, panel #1) in both untreated and μUtrn treated *mdx*^*4cv*^ mice (S4A Fig, panel #3 *vs*_panel #6). We next examined the location of FLAG-μUtrn and found that it localized in a costameric pattern with striations that were only ~0.8 μm apart (S4B and S4C Fig (*P* < 0.001)). However, μUtrn localizes in normal, 2.2 μm striations in the absence of full-length Utrn, as seen in treated *mdx*:*utrn*^-/-^ muscles (S4D Fig). Thus, high-level expression of FLAG-μUtrn in *mdx*^4cv^ mice leads to the localization of μUtrn in closely spaced striations that co-exist with the normal, FL-Utrn containing costameres.

### Assembly of the dystrophin-glycoprotein complex following μUtrn treatment

Expression of μDys has been shown to restore assembly and sarcolemmal localization of the dystrophin-glycoprotein complex (DGC) [9, 32, 85–87], so we asked whether μUtrn expression also restores DGC components in *mdx*^4cv^ muscles. This was tested utilizing the same mice described for the rAAV6-CMV-μUtrn IM injection study from Fig 2. Serial cryosections from injected gastrocnemius muscles were immunostained to detect myosin heavy chain isoforms associated with different muscle fiber types (Fig 5A–5C), utrophin (Fig 5D–5F), and the representative DGC components β-dystroglycan (β-Dgn) (Fig 5G–5I), δ-sarcoglycan (δ-Sarc) (Fig 5J–5L), α-dystrobrevin-2 (α-Db2) (Fig 5M–5O), α-1-syntrophin (α1-Syn) (Fig 5P–5R) and neuronal nitric oxide synthase (nNOS) (Fig 5S–5U).

As expected, endogenous FL-Utrn was only expressed at low levels in wild-type muscles (Fig 5D), but was up-regulated in *mdx*^4cv^ muscle fibers (Fig 5E)[88–91]. However, the relative sarcolemmal intensity of endogenous utrophin A immunostaining was highly variable between and within different fiber types (e.g., Fig 5B and 5E). In contrast, numerous myofibers within rAAV6-CMV-μUtrn IM injected *mdx*^*4cv*^ muscles exhibited intense utrophin immunostaining, with type 2b fibers displaying the highest relative intensities (e.g., Fig 5F). The DGC components, β-Dgn, δ-Sarc were broadly restored in myofiber types (Fig 5, 5I vs. 5H, and 5L vs. 5K), while α-Dbn-2, and α1-Syn were most strongly re-localized in 2b fibers (Fig 5, 5O vs. 5N, and 5R vs. 5Q), which displayed the highest expression of μUtrn. In contrast, nNOS remained relatively absent from the sarcolemma (Fig 4U vs 4S), even though α1-Syn, a DGC component with which nNOS and endogenous utrophin are known to interact [92, 93], was re-localized to the sarcolemma. This observation has been confirmed in previous work where it was shown that the α-Syn binding motif within dystrophin spectrin-like repeat-17 enables recruitment of nNOS to the sarcolemma[32, 33, 52]. These results show that μUtrn, like utrophin A, does not localize nNOS to the sarcolemma [87]. The ability of μUtrn to localize α1-Syn is presumably due to additional components of the DGC, such as α-Db isoforms that are known to interact with α1-Syn, and potentially the sarcoglycans [94]. These results thus indicate that delivery of AAV6-CMV-μUtrn to *mdx*^*4cv*^ mouse muscles localizes most, but not all, dystrophin glycoprotein-complex proteins to the sarcolemma.

### Influence of μUtrn on the structure of neuromuscular junctions

**Fig 6:**
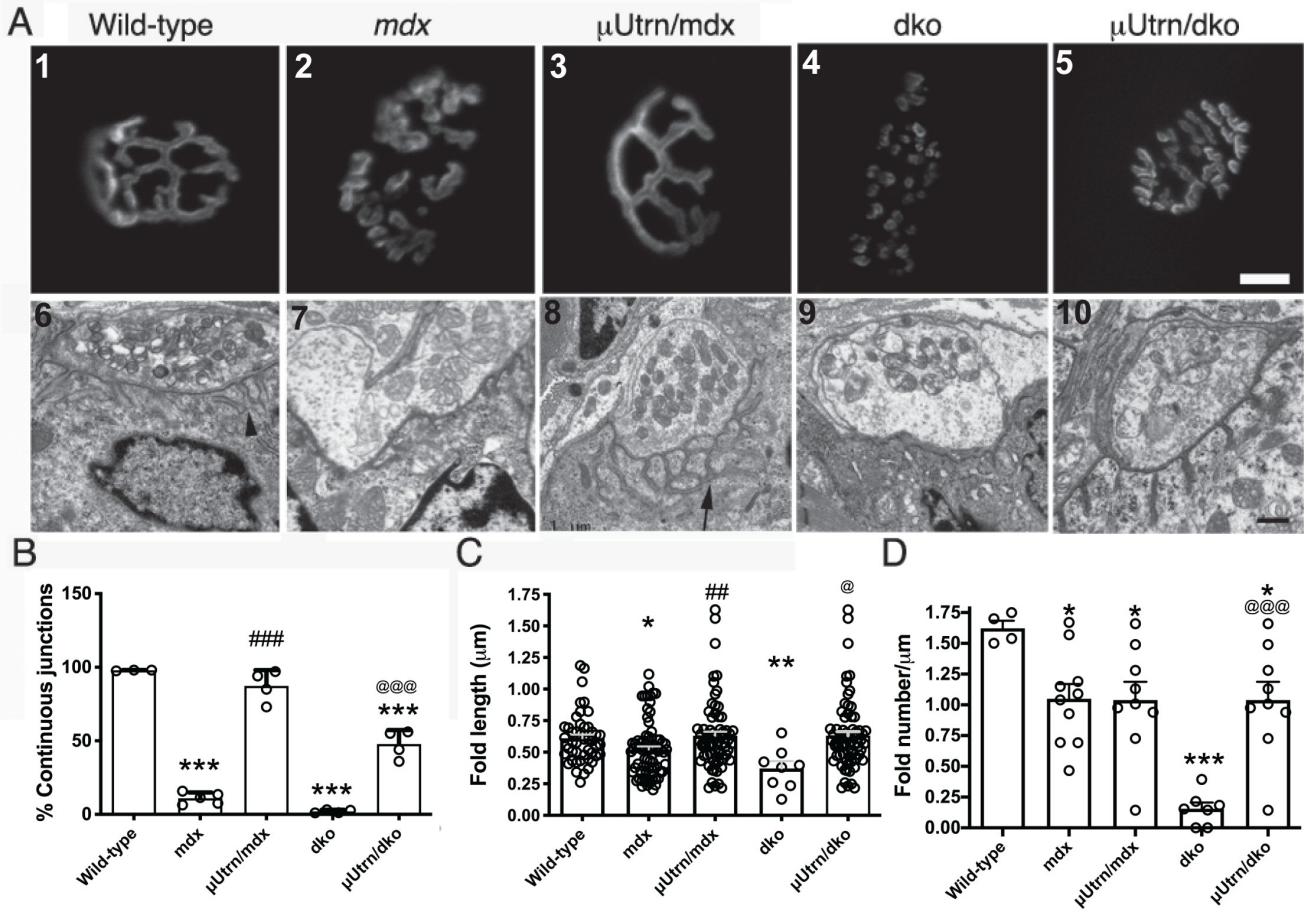
μUtrn expression partially restores the structure of neuromuscular junctions. (A) Top panel: AChR cluster visualization in the postsynaptic apparatus reveals that μUtrn expression prevents the fragmentation of synapses seen in *mdx*^*4cv*^ and *dko* muscles. Scale bar = 10 μm. Lower panel: Electron microscopic images of neuromuscular synapses reveals that the synaptic folds in wild-type mice (arrow head) are shallower and less in number in muscles from *mdx*^*4cv*^ mice and dko mice. μUtrn restores the depth, but not the number of synaptic folds. However, the synaptic folds are highly branched in the *mdx*^*4cv*^ mice treated with rAAV-μUtrn. Scale bar = 0.5 μm. (B) Mean +/- S.D. of continuous synapses. (C) Mean +/- S.D. of fold lengths in the postsynaptic apparatus. (D) Mean +/- S.D. of the number of postsynaptic folds/μm. *P < 0.05, **P < 0.001, and ***P < 0.001 compared to wild-type. ^##^*P* < 0.01 and ^###^*P* < 0.001 compared to *mdx*^*4cv*^. ^@^*P* < 0.05 and ^@@@^*P* < 0.001 compared to dko.

The acetylcholine receptor (AChR) clusters on the postsynaptic membranes of neuromuscular junctions (NMJs) in wild-type mice mature into pretzel-like profiles that exhibit generally continuous junctions (defined here as a NMJ that has three or less continuous segments) when viewed *en face* (Fig 6A, panel 1). These profiles are known to further fragment upon skeletal muscle necrosis in *mdx*^*4cv*^ and *mdx*:*utrn*^-/-^ mice [18–20, 74]. We found that μUtrn expression in gastrocnemius muscles of *mdx*^*4cv*^ and *mdx*:*utrn*^-/-^ mice prevented the formation of fragmented synapses (Fig 6A, panel #3 vs #2, and panel #5 vs #4). Importantly, the overall continuity of junctional membranes was significantly improved in μUtrn-treated muscles (Fig 6B). We also examined electron micrographs of NMJs in these mice and found, as in normal muscle fibers, that the presynaptic motor nerve terminal directly abuts the postsynaptic apparatus and that the presynaptic terminal contains folds [black arrow head, (wt) vs black arrow, (mdx+μUtrn)] that invaginate into the muscle (Fig 6A, compare panel #’s 6–10).

In wild type muscles utrophin is primarily restricted to the crests of folds whereas dystrophin is found in the troughs of folds [95, 96]. Utrophin is also restricted to the crests of folds in NMJs of *mdx* skeletal muscles (S5 Fig, panel #5). We found that the NMJs in *mdx*^*4cv*^ postsynaptic membranes have a small, but significant reduction in the length and number of folds (Fig 6C & 6D), and that μUtrn is mis-localized to the troughs of the synaptic folds in treated *mdx*^*4cv*^ muscles (S5 Fig, panel #6). μUtrn delivery restored the length of synaptic folds in *mdx*^*4cv*^ muscles to depths similar to those in wild-type muscles (Fig 6A, panels #6–8, and 6C). Interestingly however, while μUtrn did not restore the number of post-synaptic folds in *mdx*^*4cv*^ mice (Fig 6D), the folds in μUtrn-treated mice became highly branched (Fig 6A, panel #8). Expression of μUtrn did restore the number of folds in the NMJs of *mdx*:*utrn*^-/-^ muscles to the levels found in *mdx*^*4cv*^ muscles (Fig 6A panels #5 & 10, 6D).

### Influence of μUtrn on the structure of myotendinous junctions

**Fig 7:**
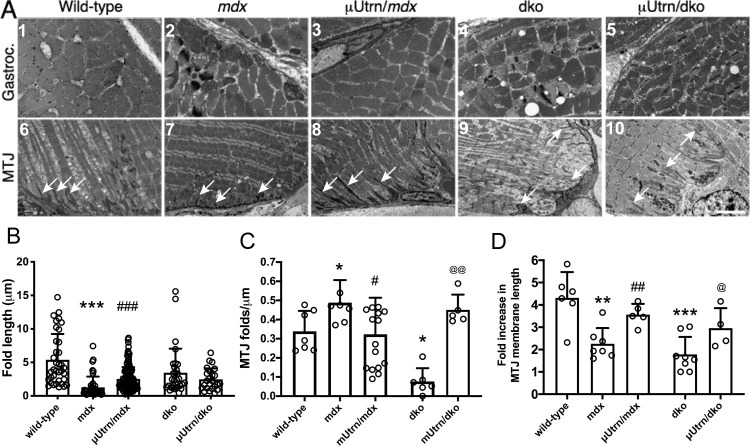
μUtrn expression partially restores the structure of myotendinous junctions without leading to ringed fiber formation. (A) Top panel: Electron microscopy of transverse sections of gastrocnemius muscles. Scale bar = 2 μm. Lower panel: Longitudinal sections of Achilles myotendinous junctions. Note the loss of folds in *mdx*^*4cv*^ and *dko* mice is partially restored by μUtrn expression. Scale bar = 5 μm. (B) Mean +/- S.D. length of folds. (C) Mean +/- S.D. fold per μm of MTJ. (D) Mean +/- S.D. increase in membrane length brought about by the MTJ folds.

Treatment of *mdx*^*4cv*^ mice with a first-generation μDys^(ΔR4-R23/ΔCT)^ leads to myotendinous strain injury and “ringbinden” or ringed fiber formation in gastrocnemius muscles [19, 26, 39]. This is associated with the presence of a polyproline motif within the ‘Hinge-2’ domain of this particular μDys [19, 26, 39]. We therefore asked whether expression of the analogous μUtrn would lead to similar deleterious effects (Fig 7). Electron micrographic analysis of transverse gastrocnemius muscle sections from untreated wt, *mdx*^4cv^, *mdx*:*utrn*^-/-^, as well as in *mdx*^4cv^ or *mdx*:*utrn*^-/-^ mice treated with μUtrn revealed no ringed fibers in any of the muscles (Fig 7A, panels #3 and #5). Thus, the polyproline motif from utrophin hinge 2 does not adversely affect muscle ultrastructure.

To assess the presence of endogenous utrophin and μUtrn within MTJs we immunostained cryosections with a C-terminal antibody (to detect endogenous utrophin) and with the FLAG antibody (to detect FLAG-μUtrn). Both types of utrophin colocalized within folds in the Achilles myotendinous junctions [S6 Fig, where each panel shows the myofiber(s) integrated into the tendon at the base of the image(s)]. Since the folds in MTJs play critical roles in reducing membrane shear stress during muscle contraction [22], we also analyzed electron micrograph sections to determine the lengths of the MTJ folds. The MTJ folds were shorter in muscles from *mdx*^*4cv*^ mice than in wild type controls, as indicated by the white arrows (Fig 7A; compare panel #6 vs #7, and Fig 7B). However, the folds were also more numerous than in controls (Fig 7C), resulting in a reduction in the total length of membrane provided by the junctional folds when compared to wild-type muscles (Fig 7D). μUtrn treatment partially restored the MTJ fold length and number in *mdx*^*4cv*^ muscles (Fig 7B and 7C); thereby essentially restoring the total length of membrane provided by the folds (Fig 7D).

## Discussion

Here we demonstrate that μUtrn improves the dystrophic pathophysiology of *mdx*^*4cv*^ muscles, as well as the maturation and maintenance of the neuromuscular and myotendinous junctions. Histologically, following μUtrn treatment there was an abrupt reduction in the number of myofibers undergoing regeneration as indicated by central nucleation. We also observed the restoration of DGC components β–Dgn and δ-Sgn, however the localization of α-Db2 and α1-Syn was skewed toward fast 2b fiber expression. Indeed, at longer time-points within our study the improvement in pathophysiology appears to primarily stem from the stable expression of μUtrn within fast 2b fibers rather than the 1a, 2a and 2d/x fiber types irrespective of the transcriptional regulatory cassette used to drive expression. These observations have potentially important implications for the treatment of DMD skeletal muscles with AAV-delivered μUtrn.

### Does steric hindrance between μUtrn and endogenous utrophin impact efficacy?

A fundamental question of this study is not only could uUtrn compensate for the lack of dystrophin, but whether uUtrn could function optimally when co-existing with the endogenous full-length utrophin. More specifically, is there potential for steric hindrance between the full-length utrophin and the smaller μUtrn that could impact the function and survival of muscle cells? Slower fiber types have long been known to have slightly higher levels of endogenous utrophin expression and this correlates with a slightly milder dystrophic phenotype in untreated *mdx* mice[67–69]. Consistent with this, in *mdx* mice, we previously found there is more endogenous utrophin in Type 1a, 2a and 2d fiber types, when compared to fast Type 2b[71]. Strikingly, we found that uUtrn is better able to prevent the dystrophic pathology in Type 2a and 2d fibers in *mdx/utrn*^*-/-*^ mice lacking both dystrophin and utrophin when compared to the same muscle fiber types in *mdx* mice (Compare S2J–S2L to S2D–S2I Fig). This phenotype was likely unique to μUtrn treatment as delivery of second-generation uDys[39] with the same vector and promoter was able to prevent the dystrophic pathology of *mdx*^*4cv*^ mice, independent of fiber-type. Furthermore, uUtrn profoundly reduced the spacing of costameres in *mdx*^*4cv*^ mice (S4 Fig) providing a unique environment for steric hindrance with full-length utrophin within the sarcolemma.

Importantly, steric hindrance requires that both the full-length utrophin and uUtrn are on the sarcolemma at the same time. Our time-course study demonstrates we targeted the majority of muscle fibers (88%) with the therapeutic at the early age when endogenous utrophin is present on the sarcolemma. We originally anticipated that the expression of μUtrn would prevent the dystrophic pathology and allow the endogenous utrophin to follow its natural time course, and dissipate from the sarcolemma as it would with dystrophin. However, Fig 2A, panels 2 & 4 shows this wasn’t the case, as low levels of the endogenous utrophin can be found on the same fibers as the μUtrn at 3 months of age. The greater levels of full-length utrophin in type 1a, 2a and 2d sarcolemma could sterically hinder the μUtrn more than in fast 2b fibers where full-length utrophin is minimal[71]. Steric hindrance between full-length utrophin and μUtrn leave the skeletal muscles more susceptible to contraction-induced injury, leading to the death of the muscle fibers and regeneration of the myotubes without the uUtrn therapeutic. This would explain why we see broad μUtrn expression and prevention of dystrophy in fast 2b fibers, but less so in other fiber types. Together, our results support an *in vivo* condition where the stoichiometry of full-length utrophin and μUtrn at the sarcolemma of skeletal muscle fibers could impact the efficacy of the therapeutic.

We also note that in a recent study with *mdx* mice and cxmd canines treated with AAV-mediated micro-utrophin demonstrated impressive efficacy at early timepoints (~2 weeks post-delivery)[46], which was also seen in dko mice[47], and to a large degree here as well. However, in the recent[46] and the present studies the functional benefit seen in *mdx*^4cv^ mice waned over time in fiber types 2A, 1A, & 2D, possibly due to competition with fl utrn. The sustained uUtrn expression in fast 2B fibers in the present study suggests the *mdx* mice did not outgrow expression as previously postulated[46], a conclusion further supported by the sustained uDystrophin expression observed for at least 8 months and up to 2 years in all fiber types in *mdx* mice, and for >1 year in DMD patients [77, 78]. The relative expression levels of fl utrn in various fiber-types and between species such as mice, dogs and humans is not well established and detailed biodistribution and time course studies will be required to understand the longevity of the therapy. Further, the canine studies were followed only for a relatively short time period, such that fiber-type expression levels might potentially wane over time.

### Neuromuscular synapse

The neuromuscular junction contains distinct structural features that ensure nerve evoked stimulation of the muscle exceeds that needed to generate an action potential in the postsynaptic membrane [97]. The presynaptic nerve terminal contains active zones where quanta of acetylcholine are released onto AChR clusters on the crests of the postsynaptic folds [95, 96]. The postsynaptic folds contain a high concentration of voltage gated sodium channels that reduce the threshold required to generate endplate currents [98]. The number of quanta reduces with repeat stimulation of the nerve during normal muscle activity [99], but the sodium channels in the folds ensure the safety of transmission is maintained [98–100]. The depth and number of folds determines the concentration of voltage gated sodium channels [19, 101]. Thus, the reduction in depth and number of folds could contribute to the functional deficits in synaptic transmission in *mdx*^*4cv*^ mice [98, 102]. Here we found that μUtrn was able to restore the depth, but not the number of fold openings in *mdx*^*4cv*^ mice. However, μUtrn expression did lead to a profound increase in fold branching. Utrophin is normally localized to the crests of folds, and dystrophin is primarily localized with the troughs of folds along with voltage gated sodium channels. Therefore, it is possible that the mislocalization of μUtrn increased the branching. In support of this hypothesis, utrophin is required to maintain the fold openings at the neuromuscular junction [103]. We previously found that miniaturized dystrophins can restore the depth and number of folds [19, 39]. Therefore, dystrophin and utrophin are both required for normal development of the synaptic folds. The neuromuscular junctions in humans are smaller than mice and the folds are deeper, suggesting the safety-factor of synaptic transmission relies more on the voltage gated sodium channels [97]. Therefore, a loss of synaptic folds and inclusive sodium channels resulting in decreased synaptic currents within the neuromuscular junctions of DMD patients could potentially reduce the neuromuscular transmission contributing to fatigue. An increase in fold branching could potentially compensate for the lack of fold openings in DMD to restore the safety of neuromuscular synaptic transmission.

### Myotendinous junction

The myotendinous junction is a major site of force transfer in skeletal muscles [22]. The folds within the MTJ reduce membrane stress under shear [22]. The depth of the folds is reduced within the Achilles MTJ in *mdx*^*4cv*^ mice and in some DMD patients [21, 23, 24, 26]. Further, the MTJ is a primary site of contraction-induced injury in *mdx*^*4cv*^ mice and some DMD patients [21, 23, 24, 26]. Treatment with μUtrn partially increased the MTJ folds in *mdx*^*4cv*^ mice and restored the sarcomere attachments in the *dko* mice. Importantly, μUtrn did not lead to chronic myotendinous strain injury or ringed fibers despite containing a polyproline site in hinge 2 [39]. Therefore, the structure and function of μUtrn likely differs from that of an analogous μDys^(ΔR4-R23/ΔCT)^ that did lead to myotendinous strain injury and ringed fiber formation when expressed in *mdx*^*4cv*^ muscles.

In conclusion, μUtrn was able to replace most functions of dystrophin at the sarcolemma, neuromuscular junctions, and myotendinous junctions of dystrophin-deficient *mdx*^*4cv*^ mice. However, we also present several lines of *in vivo* evidence consistent with steric hindrance between the full-length endogenous utrophin and uUtrn, which could impact the dystrophic pathophysiology in a myofiber type selective manner. Further studies are needed to better understand the stoichiometry of steric hindrance to predict its relevance in large animal models and humans.

## Materials and methods

### Mice and ethics statement

C57Bl/6 mice and *mdx*^*4cv*^ mice were utilized in this study. The *mdx*^*4cv*^ mice were genotyped by sequencing as previously described [104]. All Animal experiments were performed in accordance with and approval by the Institutional Animal Care and Use Committee of the University of Washington under protocol 3333–01. The UW guidelines are at least as protective as those of the National Institutes of Health, they conform to all applicable laws and regulations, they meet prevailing community standards for responsible scientific research and were applied throughout the project to ensure the humane treatment of all animals involved in the project.

### Viral vector production and injection

The μUtrn cDNA sequence was codon-optimized using GenScript (Piscataway, NJ). The rAAV6-CK8-codon optimized μUtrn and rAAV6-CMV-μUtrn (not codon optimized) expression vectors were sequenced and co-transfected with the pDGM6 packaging plasmid into HEK293 cells to generate recombinant AAV vectors comprising serotype 6 capsids. Vectors were harvested, purified, and quantitated as described previously [73]. The rAAV6-μUtrns were formulated in Hanks’ balanced salt solution and injected either directly into the gastrocnemius muscles or intravenously by retro-orbital injection at two weeks of age while the mice were anaesthetized with isofluorane.

### Histology

Muscles were frozen directly in OCT cooled in 2-methylbutane in liquid N_2_. Ten micrometer transverse sections of skeletal muscles were immunostained as previously described [71]. Briefly adjacent sections were immunostained with conjugated monoclonal antibodies to myosin heavy chain to identify fiber-types as previously described [105], N terminal utrophin antibody, C-terminal utrophin antibody (both 1:800; and kindly provided by Stanley Froehner, University of Washington, Seattle, USA), α2-laminin (1:800; SIGMA, St. Louis MO), and hematoxylin and eosin using manufacturer protocols (Electron Microscopy Sciences; Hatfeild, PA). For detecting DGC components, adjacent frozen sections were immunostained with α-dystrobrevin 2 (1:1000), α1-syntrophin (1:500; the latter two antibodies were kind gifts from Stanley Froehner), β-dystroglycan (1:100; BD Biosciences, San Jose, CA), δ-sarcoglycan (1:40; Leica Biosystems, Buffalo Grove, IL), or nNOS (1,100; Invitrogen, Carlsbad, CA). For detecting AChRs, cryosections we incubated in α-bungarotoxin conjugated to TRITC (1,800; Invitrogen, Carlsbad, CA). All fluorescent immunostained sections were coverslipped with ProLong Gold mounting medium containing DAPI (Invitrogen, Carlsbad, CA). Sections were imaged with either a Leica SP5 confocal or an Olympus SZX16 dissection fluorescent microscope.

### Immunoblotting

Western blots were performed on whole muscle lysates as previously described [26]. Briefly, the gastrocnemius muscles treated *mdx*^*4cv*^ muscles and contralateral controls (n = 4) were ground in liquid N_2_ and homogenized in extract buffer (50 mM Tris-HCl, 150 mM NaCl, 0.2% SDS, 24 mM Na deoxycholate, 1% NP40, 47.6 mM Na Fluoride, 200 mM Na Orthovanadate, Roche, Basel, CH). Protein concentration of whole muscle was determined by Coomassie Plus Bradford Assay (Thermoscientific, Rockford, IL). Equal amounts of protein (20 μg) were resolved on a 4–12% SDS polyacrylamide gel. The blots were incubated in N-terminal anti-utrophin (1:1000; kind gift from Stanley C. Froehner) overnight at 4°C. The GAPDH antibody (1:50,000; Sigma, St. Louis, MO) was used as a loading control as its expression was unchanged when comparing the treated and untreated *mdx*^*4cv*^ muscles. The primary antibodies were detected with IgG HRP secondary antibodies (1:25,000; Jackson ImmunoResearch Labs). The blots were developed with ECL plus (Thermoscientific, Rockford, IL) and scanned with the Storm 860 imaging system (GE Healthcare Lifesciences, Piscataway, NJ). The band intensity was measured using Image J software (NIH).

### Real time PCR

To isolate the RNA, approximately 20μg of gastrocnemius muscle previously ground by mortar and pestle in liquid N_2_ was used to extract total RNA following manufacturer’s instructions (TRI Reagent, Molecular Research Center, Inc. Cincinnati, OH). The pelleted RNA was suspended in 50 μl nuclease free elution solution (Ambion Inc., Austin, TX). Five μg of total RNA was treated with Turbo DNA-free (Ambion Inc., Austin, TX) in order to remove trace amounts of contaminating DNA. The DNAase Treated RNA (0.5μg) was diluted to 8μl with nuclease free water followed by use of the SuperScript III First-Strand Synthesis kit (Invitrogen, Carlsbad, CA) to generate cDNA. Subsequently 2μl of the cDNA was used for qPCR with utrophin primer-probe sets. The mouse utrophin oligonucleotide sequences were: Forward 5’- ACCAGCTGGACCGATGGA-3’, Reverse 5’- CTCGTCCCAGTCGAAGAGATCT-3’, Probe 5’-6FAM- CGTTCAACGCCGTGCTCCACC-3’-BHQa1-Q. The primer sequences for the μUtrn H2-R22 unique junction were Forward 5’-GCGATAACCTGGAGACCTGAAG-3’, reverse 5’-TTTATTACTAGCCACCGGTATCGAT-3’, probe 6FAM-ATTCATCCGGCCAACCAATGTTCTCG. As a reference gene the oligonucleotide set was used to target the mouse Ywhaz gene sequence (Tyrosine 3-monooxygenase; [106]): Forward 5’- GCTGGTGATGACAAGAAAGGAAT-3’, Reverse 5’- GGTGTGTCGGCTGCATCTC-3’, Probe 5’-6FAM-TGGACCAGTCACAGCAAGCATACCAAGA-3’-BHQa1-Q.

### Muscle fiber areas

The muscle fiber areas were quantitated for each fiber-type using the FIJI Open Source image processing software package based on ImageJ, as previously described [71].

### Skeletal muscle physiology

The tibialis anterior muscle physiology was performed as previously described [37, 39, 47, 73, 107].

### Electron microscopy

The electron microscopy was performed on transverse and longitudinal sections of the gastrocnemius muscles as previously described [26]. The junctional fold number and lengths were measured from N = 4 mice at 3 months of age in mdx mice treated with the rAAV6-CK8-μUtrn and the contralateral control using FIJI computer program. The counts represent the fold numbers and lengths from all fibers.

### Quantitation of neuromuscular synapses

Neuromuscular synapses were analyzed in wholemount immunostained teased muscle fibers and quantitated as previously described [19, 39]. Synapses were quantitated from N = 4 *mdx*^*4cv*^ or dko muscles treated with rAAV6-CK8-mUtrn or rAAV6-CMV-μUtrn respectively.

### Statistics

The data were compared using a one-way ANOVA with a Tukey post-test that compares all data sets with a Student’s t-test. All data analyses were performed using the PRISM software.

## Supporting information

S1 Fig (A.) μUtrn expression reduces the number of fibers undergoing regeneration. The number of developmental myosin heavy chain positive fibers in the gastrocnemius muscles are displayed at 4 months of age. Data are shown as mean +/- S.D. **P < 0.01 compared to wild-type. ^##^*P* < 0.01.
(TIF)

S2 Fig Fiber-type expression of μUtrn expression is influenced by expression of endogenous full-length utrophin. A) μUtrn is present in all fiber-types 2 weeks after vector administration at similar levels each approaching ~90%; B) corresponding myofiber typing for the 2 week time point; and C) Quantification of the proportion of fiber types expressing μUtrn (Mean +/- S.D.). D) μUtrn is unable to prevent necrosis in most 1a, 2a and 2d/x fiber types at 3 months of age. E) Representative fiber typing; F) Mean +/- S.D. proportion of μUtrn-positive fiber types. ***P < 0.001 compared to the fast 2b fibers at 3 months of age. G) μUtrn is predominantly expressed in the fast 2b fibers when driven by the CMV promoter. H) Representative fiber typing; I) Mean +/- S.D. proportion of μUtrn positive fiber types. ***P < 0.001 compared to the fast 2b fibers at 4 months of age. J) μUtrn was not selective for the fast 2b fibers in *mdx*^*4cv*^:utrophin double knockout (*dko*) mice. K) Representative fiber typing; & L) Mean +/- S.D. proportion of μUtrn positive fiber types. *P < 0.05 compared to the 2a, 2b and 2d fiber types at 4 months of age. Scale bar = 100 μm.
(TIF)

S3 Fig Intravenous administration of rAAV6-CK8-microdystrophin^(ΔH2-21/ΔCT+H3)^ vectors to *mdx*^*4cv*^ mice results widespread expression & increased muscle function at 9 months post-administration. In comparison to untreated *mdx*^*4cv*^, the gastrocnemius muscles of μDys treated *mdx*^*4cv*^ mice exhibited (A) increased force generating capacity; (B) increased specific force (sPo); (C) decreased susceptibility to eccentric contraction-induced injury; (D) increased recovery force generation; and (E) Display of broad immunostaining for dystrophin and (F.) corresponding adjacent H&E. Data are shown as mean +/- S.D. **P < 0.01 compared to wild-type. ^##^*P* < 0.01, ^###^*P* < 0.001, compared to *mdx*^*4cv*^. sPo, specific force; WT, wild type; hematoxylin & eosin, H&E. Scale bar = 50 μm.
(TIF)

S4 Fig Influence of μUtrn localization on the costameres. (A) Utrophin localizes in a rectilinear pattern with α-sarcomeric actin in *mdx*^*4cv*^ and rAAV-μUtrn treated muscles (S4 Fig, panels 3 & 6). Note however, the prominent utrophin localization between the large costameric striations. Scale bar = 10 μm. (B) Localization of μUtrn with the FLAG antibody revealed the costameric striations to be very close together. Scale bar = 10 μm. C) Immunostaining of μUtrn with the utrophin A antibody reveals the costameric striations in *dko* mice treated with AAV6-CMV-μUtrn. Scale bar = 10 μm. Mean +/- S.D. distance between the costameric striations in *mdx*^*4cv*^ controls and the FLAG-μUtrn expressing muscles. ^###^*P* < 0.001.
(TIF)

S5 Fig μUtrn localization within neuromuscular synapses of *mdx* mouse muscles. Note that utrophin (green) localizes on the crests of the folds in *mdx* mice (arrow in top panel inset). However, FLAG-μUtrn was found within the folds (arrow in lower panel inset). Note also the lack of subsynaptic nuclei (blue, DAPI) in the *mdx* myofiber, but not the μUtrn/mdx myofiber. This *mdx* myofiber (top panel) has regenerated as revealed by the centrally-located nucleus. α-bungaratoxin (αBTX) staining is shown in red. Scale bar = 10 μm.
(TIF)

S6 Fig μUtrn within the *mdx* myotendinous junctions. Note that utrophin and FLAG-μUtrn (green) were found in the folds (merged panel, arrows). α2-laminin is shown in red, while DAPI is shown in blue. Scale bar = 20 μm.
(TIF)
