# Supplementary figures and images for: Microutrophin expression in dystrophic mice displays myofiber type differences in therapeutic effects
Source: PLoS Genet. 2020 Nov 11;16(11):e1009179. doi: 10.1371/journal.pgen.1009179 (PMC7682874; doi:10.1371/journal.pgen.1009179)

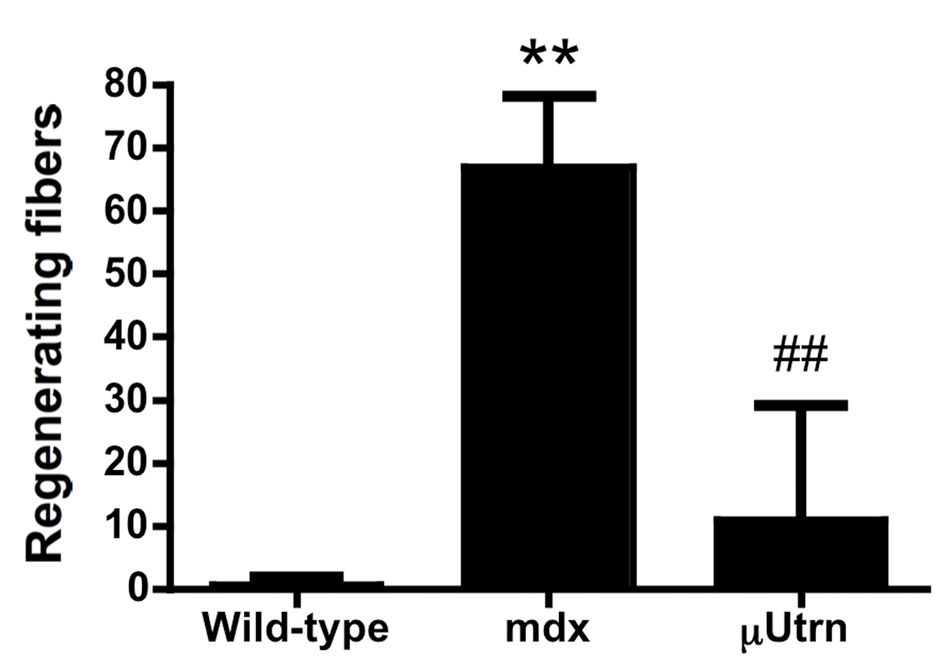

Supplement: S1 Fig — (A.) μUtrn expression reduces the number of fibers undergoing regeneration. The number of developmental myosin heavy chain positive fibers in the gastrocnemius muscles are displayed at 4 months of age. Data are shown as mean +/- S.D. **P < 0.01 compared to wild-type. ##P < 0.01. (TIF) [file pgen.1009179.s001.tif]

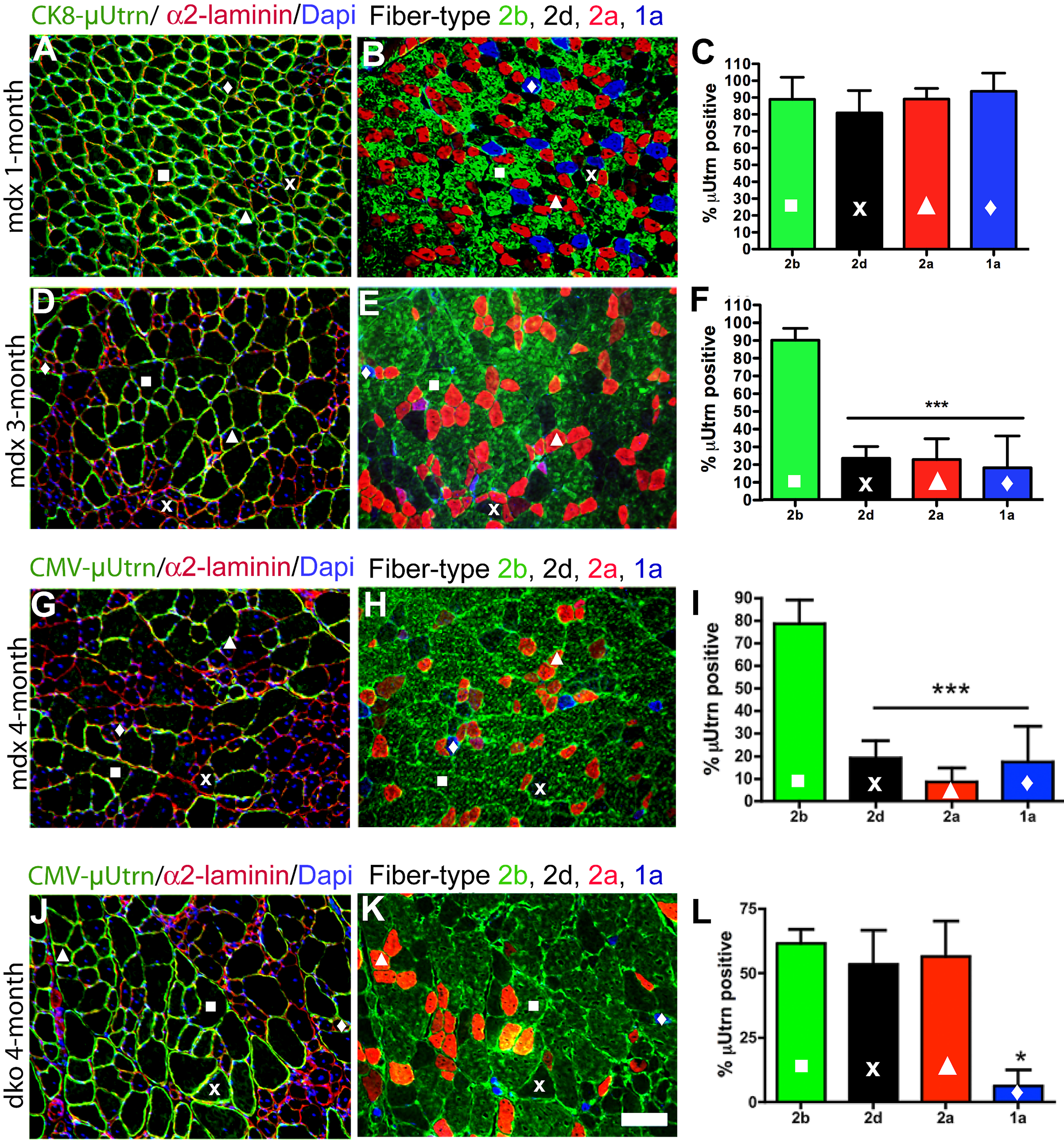

Supplement: S2 Fig — A) μUtrn is present in all fiber-types 2 weeks after vector administration at similar levels each approaching ~90%; B) corresponding myofiber typing for the 2 week time point; and C) Quantification of the proportion of fiber types expressing μUtrn (Mean +/- S.D.). D) μUtrn is unable to prevent necrosis in most 1a, 2a and 2d/x fiber types at 3 months of age. E) Representative fiber typing; F) Mean +/- S.D. proportion of μUtrn-positive fiber types. ***P < 0.001 compared to the fast 2b fibers at 3 months of age. G) μUtrn is predominantly expressed in the fast 2b fibers when driven by the CMV promoter. H) Representative fiber typing; I) Mean +/- S.D. proportion of μUtrn positive fiber types. ***P < 0.001 compared to the fast 2b fibers at 4 months of age. J) μUtrn was not selective for the fast 2b fibers in mdx4cv:utrophin double knockout (dko) mice. K) Representative fiber typing; & L) Mean +/- S.D. proportion of μUtrn positive fiber types. *P < 0.05 compared to the 2a, 2b and 2d fiber types at 4 months of age. Scale bar = 100 μm. (TIF) [file pgen.1009179.s002.tif]

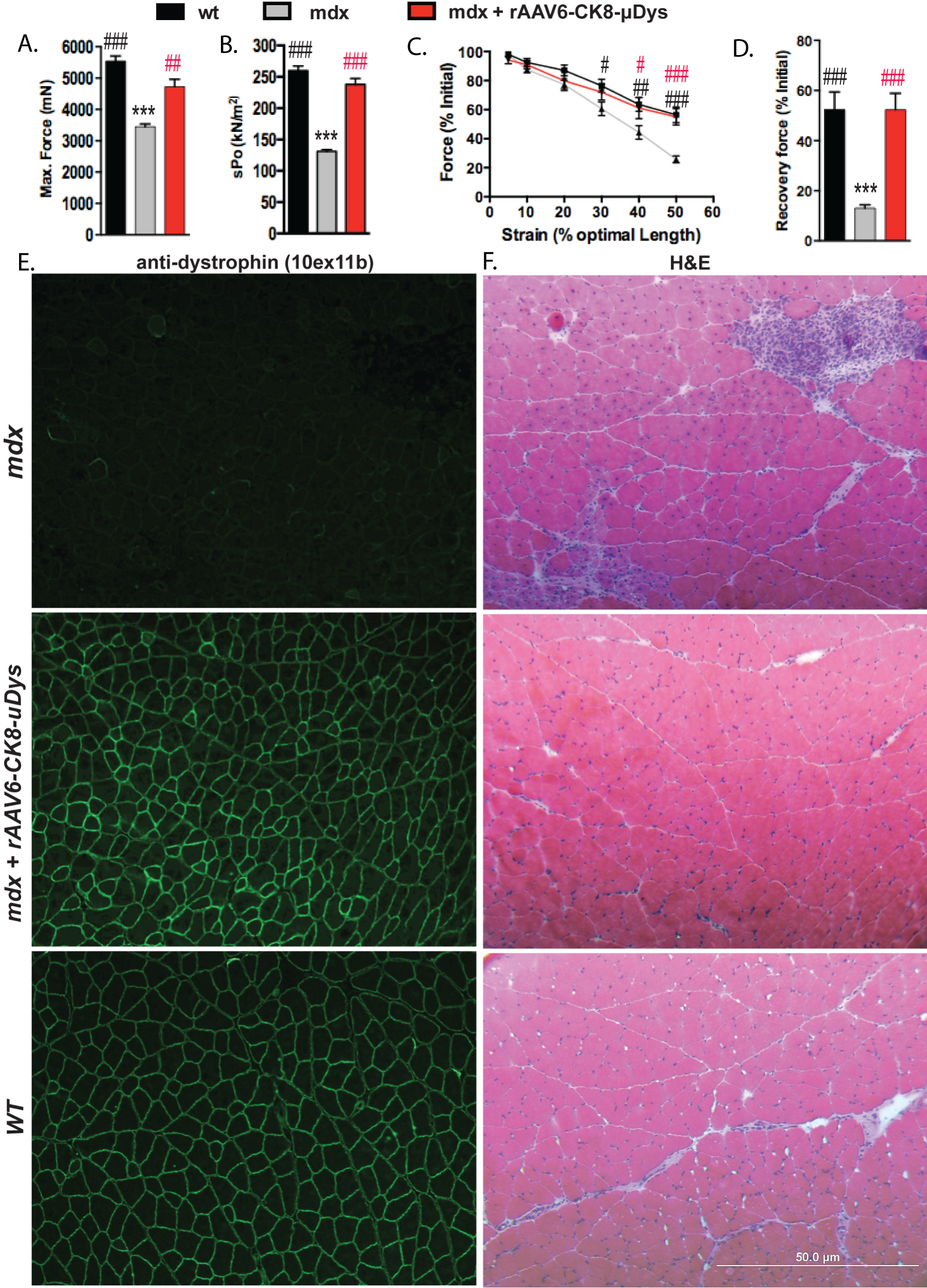

Supplement: S3 Fig — In comparison to untreated mdx4cv, the gastrocnemius muscles of μDys treated mdx4cv mice exhibited (A) increased force generating capacity; (B) increased specific force (sPo); (C) decreased susceptibility to eccentric contraction-induced injury; (D) increased recovery force generation; and (E) Display of broad immunostaining for dystrophin and (F.) corresponding adjacent H&E. Data are shown as mean +/- S.D. **P < 0.01 compared to wild-type. ##P < 0.01, ###P < 0.001, compared to mdx4cv. sPo, specific force; WT, wild type; hematoxylin & eosin, H&E. Scale bar = 50 μm. (TIF) [file pgen.1009179.s003.tif]

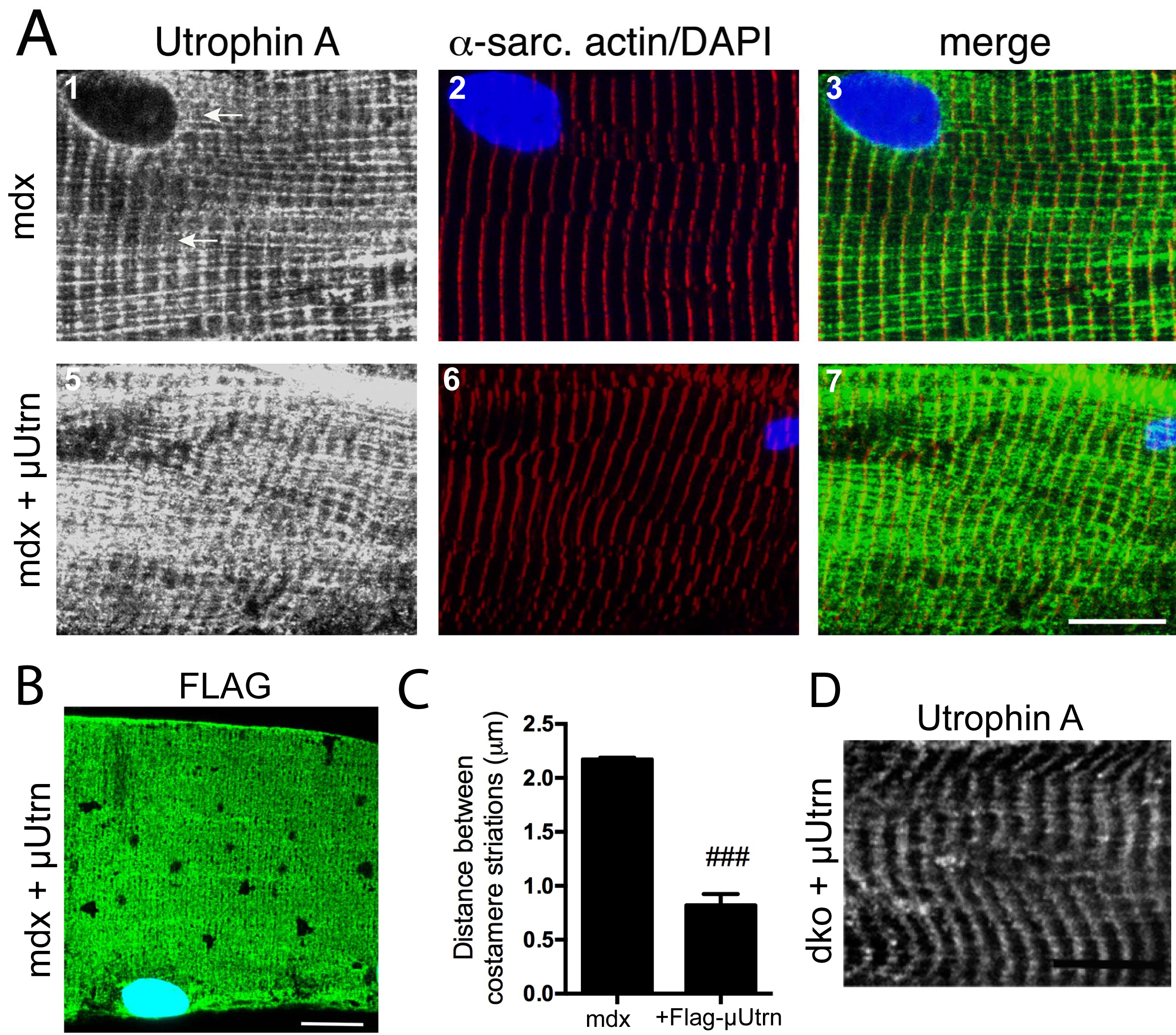

Supplement: S4 Fig — (A) Utrophin localizes in a rectilinear pattern with α-sarcomeric actin in mdx4cv and rAAV-μUtrn treated muscles (S4 Fig, panels 3 & 6). Note however, the prominent utrophin localization between the large costameric striations. Scale bar = 10 μm. (B) Localization of μUtrn with the FLAG antibody revealed the costameric striations to be very close together. Scale bar = 10 μm. C) Immunostaining of μUtrn with the utrophin A antibody reveals the costameric striations in dko mice treated with AAV6-CMV-μUtrn. Scale bar = 10 μm. Mean +/- S.D. distance between the costameric striations in mdx4cv controls and the FLAG-μUtrn expressing muscles. ###P < 0.001. (TIF) [file pgen.1009179.s004.tif]

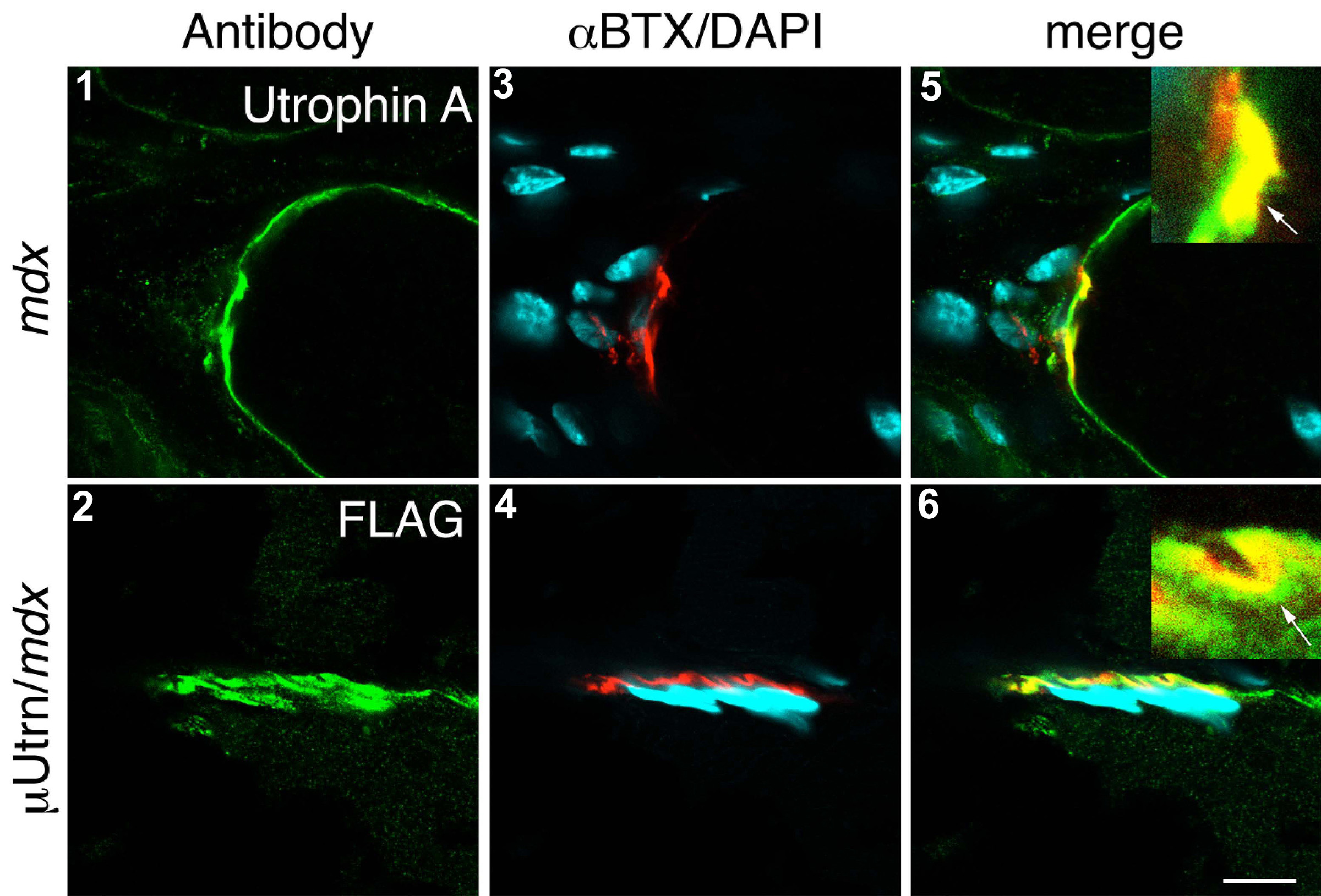

Supplement: S5 Fig — Note that utrophin (green) localizes on the crests of the folds in mdx mice (arrow in top panel inset). However, FLAG-μUtrn was found within the folds (arrow in lower panel inset). Note also the lack of subsynaptic nuclei (blue, DAPI) in the mdx myofiber, but not the μUtrn/mdx myofiber. This mdx myofiber (top panel) has regenerated as revealed by the centrally-located nucleus. α-bungaratoxin (αBTX) staining is shown in red. Scale bar = 10 μm. (TIF) [file pgen.1009179.s005.tif]

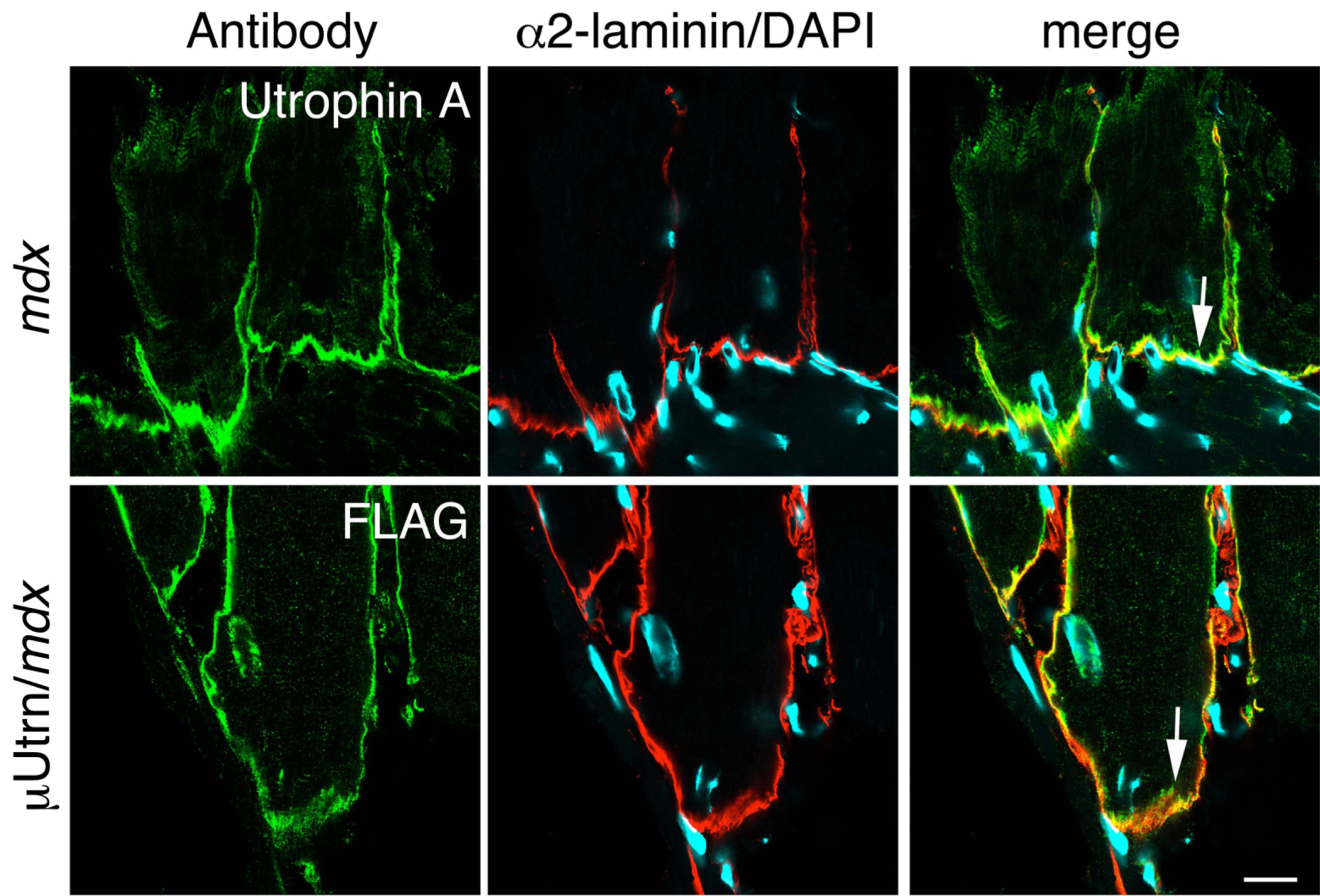

Supplement: S6 Fig — Note that utrophin and FLAG-μUtrn (green) were found in the folds (merged panel, arrows). α2-laminin is shown in red, while DAPI is shown in blue. Scale bar = 20 μm. (TIF) [file pgen.1009179.s006.tif]
